# Resilience to AD pathology in Top Cognitive Performers

**DOI:** 10.3389/fnagi.2024.1428695

**Published:** 2024-07-11

**Authors:** Elena Nicole Dominguez, María M. Corrada, Claudia H. Kawas, Craig E. L. Stark

**Affiliations:** ^1^Department of Neurobiology and Behavior, University of California, Irvine, Irvine, CA, United States; ^2^Department of Neurology, University of California, Irvine, Irvine, CA, United States; ^3^Department of Epidemiology, University of California, Irvine, Irvine, CA, United States

**Keywords:** Top Cognitive Performer, successful aging, SuperAger, oldest-old, amyloid

## Abstract

Successful cognitive aging is often thought to result from resistance to the accumulation of pathology, resilience to the effects of pathological accumulation, or some combination of the two. While evidence for resilience has been found in typical aging populations, the oldest-old provide us with a unique window into the role of pathological accumulation in impacting cognition. Here, we aimed to assess group differences in measures of amyloid and tau across older age groups using data from the Alzheimer's Disease Neuroimaging Initiative (ADNI age: 60–89) and *The 90*+ *Study* (age: 90–101). Additionally, using the ADNI dataset, we performed exploratory analyses of regional cingulate AV-45 SUVRs to assess if amyloid load in particular areas was associated with Top Cognitive Performance (TCP). Consistent with the literature, results showed no group differences in amyloid SUVRs both regionally and in the whole cortex. For tau with AV-1451, we also observed no differences in Braak composite SUVRs. Interestingly, these relationships persisted in the oldest-old. This indicates that Top Cognitive Performance throughout aging does not reflect resistance to amyloid and tau burden, but that other mechanisms may be associated with protection against amyloid and tau related neurodegeneration.

## Introduction

Successful aging is typically attributed to either resistance and resilience. Though a common core feature of both is the maintenance of stellar cognition in old age, resistant individuals show few if any neurodegenerative brain insults, while resilient individuals maintain their cognition even in the presence of said neuropathology and brain atrophy (Rowe and Kahn, 1997; Montine et al., 2019). To explore both resistance and resilience, researchers have studied the brains of specialized cohorts compared to normal-for-age individuals to determine what structural and pathological characteristics contribute to preserved cognition in older adults. The relationship among amyloid plaques, tau neurofibrillary tangles, and cognitive decline in Alzheimer's disease (AD) and typical aging have been a primary focus of research into resilience and reserve.

It is certainly clear that amyloid and tau are strongly linked to pathological processes associated with Alzheimer's disease and that it is associated with cognitive impairment (Rentz et al., 2010; Hedden et al., 2013). The presence of amyloid plaques has been linked to apoptosis, synaptic loss, altered calcium homeostasis, and disruptions in various cellular processes (Carrillo-Mora et al., 2014; Reiss et al., 2018). Similar toxic effects, such as synaptic dysfunction are seen in the presence of tau tangles and it was shown that eliminating tau through knockout mice protected against amyloid induced toxicity, alluding to its mediating effects (Leroy et al., 2012; Bloom, 2014). Early post mortem studies of AD showed that the accumulation of tau tangles was positively associated with the magnitude of cognitive decline seen in both individuals with dementia and normal cognition (Arriagada et al., 1992a,b). Furthermore, the standard Braak staging of progression of AD (Braak and Braak, 1991) is positively associated with memory impairment and conversion to dementia (Riley et al., 2002; Cho et al., 2016; Schöll et al., 2016). Early PET imaging studies have shown that individuals with dementia displayed greater amounts of amyloid deposition when compared to cognitively normal participants (Klunk et al., 2004) and several studies have highlighted the cingulate in particular as a being especially sensitive (Li et al., 2008; Chetelat et al., 2012). Earlier reports by *The 90*+ *Study* revealed that tau and amyloid continue to be significantly associated with all-cause dementia in the oldest old (Robinson et al., 2011). For example, amyloid load in 13 participants was significantly correlated with global cognition and memory, and those deemed as amyloid positive exhibited steeper declines over a 1.5 year follow up (Kawas et al., 2013), suggesting that increased amyloid can be used as a surrogate marker for rapid cognitive decline in the oldest-old.

However, amyloid and tau pathology also appear in successful aging and cognitively normal individuals, leading to the question of how they differentially contribute to any changes or preservation seen in cognition. In particular, it has become clear that there are a large number of older adults carrying an amyloid burden without clear cognitive decline. For example, in a large cross-sectional study of 7,105 individuals with normal cognition, 25.6% met criteria for amyloid abnormality (Jansen et al., 2022). AD-related lesions accumulate in the brain years before cognitive deficits (Morris and Price, 2001; Rowe et al., 2007), with longitudinal studies showing both concentrations of amyloid-beta in the CSF and amyloid deposition measured by PET appearing 25 and 15 years before symptom onset, respectively (Bateman et al., 2012). Whether these findings represent a preclinical state and burden (Morris and Price, 2001; Rowe et al., 2007), or whether they represent resilience (or both), has not been clear.

Though evidence suggests that amyloid and tau are significant contributors to decline in most models of aging, less is known about whether highly successful cognitive aging is associated with either the absence of their accumulation (resistance) or with cognitive preservation despite the accumulation of disease-related insults (resilience). A substantial amount of the literature suggests that successfully aging individuals demonstrate resilience in that they do not differ from other cognitively typical older controls in amyloid positivity or load, despite maintaining higher than typical levels of cognition. For instance, post-mortem investigations conducted as part of The 90+ Study revealed that Superior Global Cognitive Performers (SGCP), who consistently maintained a Mini-Mental State Examination score of ≥ 28 during their last visits before death, did not differ from non-SGCP in measures of Alzheimer's Disease Neuropathological Change (Biswas et al., 2023). Likewise, in younger cohorts, despite demonstrating a 69%−73% reduced risk of disease progression, SuperAgers exhibited a burden of amyloid similar to that of cognitively normal peers of their age (Dang et al., 2019). The APOE (Apolipoprotein E) gene is significantly associated with cognitive function and neurodegenerative diseases, particularly Alzheimer's disease. Those who carry the ε4 allele are more likely to experience impairments in memory and executive function, two hallmark domains in the classification of successful aging. Despite this, some studies have found that SuperAgers display similar rates of the APOE ε4 allele as typical older adults (Dekhtyar et al., 2017; Harrison et al., 2018; Dang et al., 2019; Spencer et al., 2022). Additionally, studies using blood biomarkers of dementia also showed no difference in measures of amyloid or tau (Garo-Pascual et al., 2023). These data suggest that intact cognitive performance does not reflect resistance to AD-related pathology, despite the notion that increased amyloid deposition drives cognitive decline.

Contrary to this conclusion, though, some literature suggests that successfully aging individuals may indeed resist the accumulation of Alzheimer's disease related pathology. For example, SuperAgers have been shown to exhibit lower tau burden in the inferior temporal lobe and precuneus when compared to normal agers (Hoenig et al., 2020) and lower neurofibrillary tau counts in the pregenual anterior cingulate anterior midcingulate cortex when compared to older controls in a small subset of participants (Gefen et al., 2015). An early post-mortem study of five SuperAger cases and five controls found that three of the five 80+ year old SuperAgers were determined to be Braak stage of 0, I, or II while only one control case exhibited a similar low pathology (Rogalski et al., 2013). When examining a cohort of younger 70 year old SuperAgers based on 4 separate definitions using domains such as memory, executive function, and attention (Pezzoli et al., 2024), researchers found that each classification of SuperAger exhibited lower levels of tau accumulation in the entorhinal cortex. As for studies of amyloid burden, 70 year old “supernormals”, defined by stringent criteria based on 5-year maintenance of episodic memory and executive functioning, exhibited lower amyloid burden in the right isthmus cingulate following a brain wide ROI-analysis, suggesting the possibility of regional differences rather than global (Baran et al., 2018).

Given these conflicting relationships between amyloid and successful aging and the sparsity of *in vivo* tau/SuperAging research, we sought to assess group differences in amyloid and tau in Top Cognitive Performers. Additionally, these relationships are understudied in the oldest-old, leaving us with questions of how these associations present in advanced aging. Thus, our aim is to assess group differences in amyloid and tau burden via PET, using datasets from the Alzheimer's Disease Neuroimaging Initiative (ADNI) and 90+ Study.

## Data sources and methods

### Alzheimer's disease neuroimaging initiative (ADNI)

#### Participants

**Figure 1:**
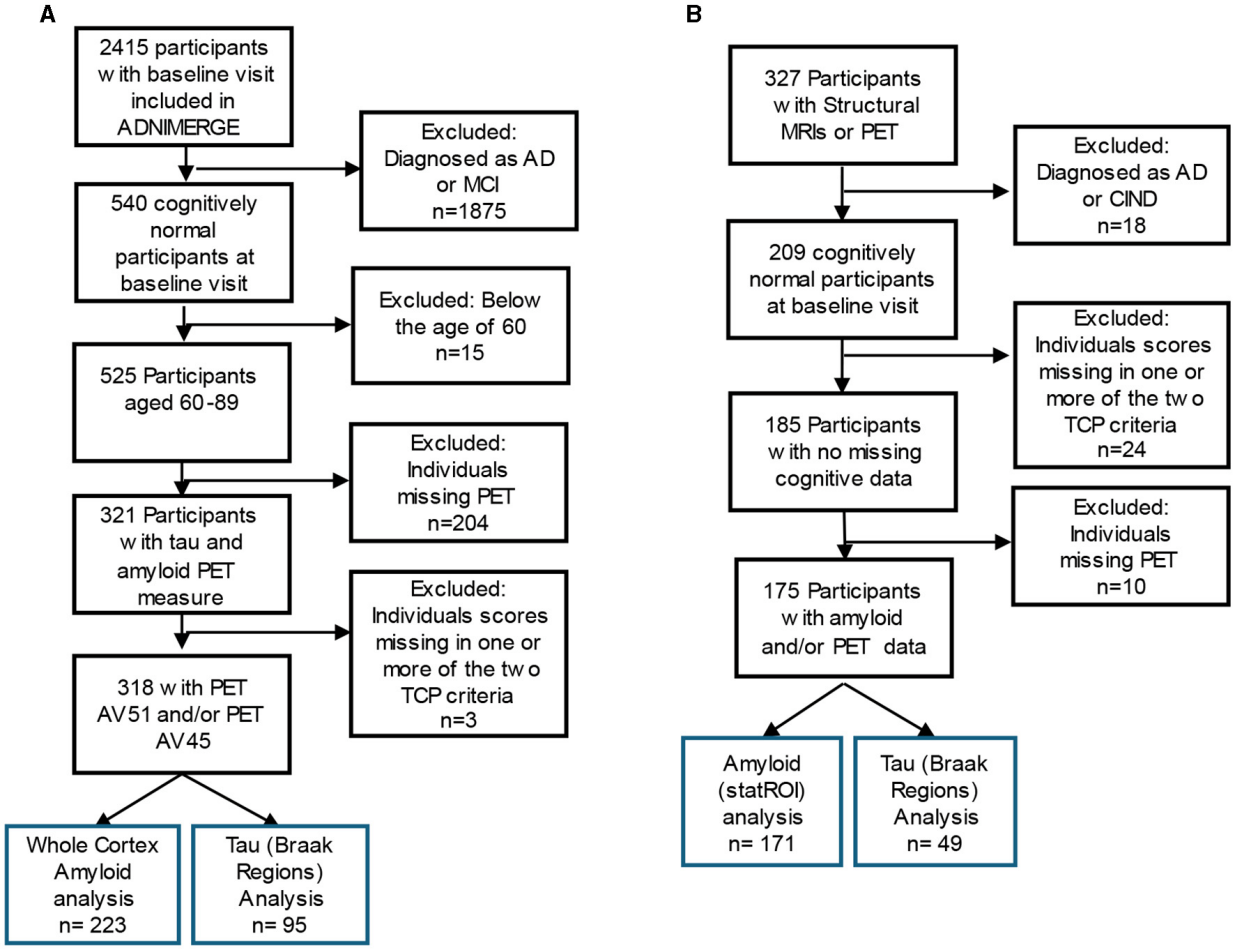
Inclusion flow chart for **(A)** ADNI and **(B)** The 90+ Study Participants. Blue box reflects the participants included in the final analysis of top cognitive performers (TCP).

The data used here were obtained from the ADNI database (adni.loni.usc.edu), downloaded on June 19, 2022. Using ADNIMERGE, which includes 2,415 participants, we restricted this analysis to baseline visits. Individuals were required to be 60 years old and above (60–89 years old) and having at least one 18F-AV1451 or [18F]-AV45 PET scan. Additionally, participants were required to have a diagnosis indicating cognitively normal, as determined by absence of depression, mild cognitive impairment, or dementia. Individuals who were missing data in any of the criteria variables, described below, were excluded from the analyses. As a result, 318 individuals were selected as a subset of the larger ADNI cohort (inclusion criteria found in Figure 1A).

#### Neuropsychological criteria for group inclusion

Previous studies of successfully aging cohorts have used neuropsychological tests with specific criteria based either on performance being consistent with a younger population or with performance being atypically high for their age group. Following the latter, TCPs were required to be in the top 50th percentile for both the Wechsler Memory Scale-revised Logical Memory IIA-Delayed Recall (WMS-R IIA) and Trails Making Test- Part B (Trails-B). The WMS-R IIA tests verbal and visual modalities and asks participants to recall units of a story after a 15 min delay (Wechsler, 1987). Trails-B engages executive function and processing speed by asking the participant to draw a line that connects an ordered progression of alternating letters and numbers (e.g. 1 – A – 2 – B – 3 – C…) as quickly as possible (Tombaugh, 2004). All individuals that did not fit these criteria were classified as non-Top Cognitive Performers (non-TCP). This resulted in TCP rates of 24–27% in the various populations.

#### Imaging data

In the ADNI cohort, all imaging data were drawn from ADNI-supplied summary metrics. Details on ADNI's collection and processing methods are described elsewhere (Landau and Jagust, 2015; Landau et al., 2021, 2022). For the AV45 (Florbetapir) data, the recommended (Landau et al., 2022) SUMMARYSUVR_WHOLECEREBNORM measure (aka AV45_bl) was used in the primary analysis as this merges the fontal, anterior cingulate, precuneus, and parietal cortex relative to the cerebellum. Here, a threshold of 1.1 was used for determining positivity and values without partial volume correction were used. For the AV1451 (flortaucipir) data, we used the inferior cerebellum as a reference region as recommended (Landau et al., 2021) for all Braak composite SUVRs provided by ADNI. Examples of regions included in each composite score are as follows: Braak 1 (entorhinal cortex), Braak 3 (parahippocampal, fusiform, lingual, and amygdala), Braak 4 (middle temporal, caudal anterior cingulate, rostral anterior cingulate, posterior cingulate, isthmus cingulate, insula, inferior temporal, temporal pole), Braak 5 (superior frontal, lateral orbitofrontal, caudal middle frontal, rostral middle frontal, etc.) and Braak 6 (pericalcarine postcentral, cuneus, precentral, and paracentral) (see full list in Landau et al., 2021). Braak 2 SUVR data was not included in this analysis as ADNI states this region was contaminated by off-target binding in the choroid plexus.

### The 90+ study

#### Participants

185 individuals from the larger *The 90*+ *Study* cohort were included (Figure 1B) and all data were provided by The 90+ Study. The 90+ Study, established in 2003, is an ongoing longitudinal investigation of aging and dementia in individuals aged 90 and above, consisting of the survivors of the Leisure World Cohort Study (Kawas, 2008). Participants were selected based on the availability of PET measures of amyloid (StatROI) and tau (BakerBraak1_2, BakerBraak3_4, BakerBraak5_6) and a cognitively normal diagnosis at baseline visit. Cognitively normal was determined by *The 90*+ *Study* and refers to a primary diagnosis, determined by neurological examiners, where an individual is deemed to have normal cognition, absent of significant impairment in any cognitive domains, and able to complete Instrumental activities of daily living (IADL). Individuals who contained missing data in any of the criteria variables were excluded from the analyses.

#### Neuropsychological criteria for group inclusion

Following NACC and ADNI TCP criteria, the 90+ TCP individuals were required to perform at or above the top 50^th^ percentile for their age group on the long-delay recognition portion of the California Verbal Learning Test—short form (CVLT) and at or above the top 50th percentile on completion time for their age group in the Trails-B (Melikyan et al., 2019). All other individuals that did not fit these criteria were classified as non-Top Cognitive Performers (non-TCP).

#### Imaging data

As with the ADNI dataset, amyloid estimates were generated using AV45 (Florbetapir) tau estimates were generated using AV-1451 (Flortaucipir). Both were fully processed summary data were provided by *The 90*+ *Study*. Details on their processing are available elsewhere (Lau et al., 2021). Note that amyloid burden in *The 90*+ *Study* (StatROI) is quantified slightly differently than in ADNI's standard approach, here as standard uptake SUVR of posterior cingulate and precuneus regions relative to an eroded cerebral white matter mask as a reference region. These regions were chosen by The 90+ because their mean SUVR distributions were able to distinguish cognitively normal individuals from the impaired (Lau et al., 2021). This is similar to the ADNI “composite ROI” normalization procedure which relies upon eroded white matter (but extends into the brainstem and cerebellum). As such, the threshold for amyloid positivity here, provided by The 90+ Study, is 0.76 and quite similar to the ADNI threshold of 0.79 using the composite ROI approach.

#### Statistical analysis of PET study participants and SUVR

For both datasets, statistical analyses were performed using Jasp and the Statsmodels (https://www.statsmodels.org/) library in Python. ANCOVAs were used to evaluate group differences in amyloid and tau levels. Additionally, unpaired *t*-tests were used to evaluate differences in continuous variables (age, education-ADNI, and neuropsychological performance) and Fisher's exact test to evaluate gender distribution, across the two subject groups.

## Results

### Demographics and neuropsychological performance at baseline

In ADNI, there were 223 individuals who fit our inclusion criteria for amyloid analyses and 95 individuals for tau analyses (Table 1). For both sets of participants, unpaired t-tests revealed TCP vs. non-TCP differences in education [amyloid: t_(221)_ = 2.72, *p* < 0.01 and tau: t_(93)_ = 3.33, *p* < 0.005], mean performance in memory [amyloid: t_(221)_ = 11.3, *p* < 0.0001 and tau: t_(93)_ = 7.2, *p* < 0.0001], and mean performance in executive function [amyloid: t_(221)_ = 5.8, *p* < 0.0001 and tau: t_(93)_ = 3.9, p < 0.0005]. The latter two are, of course, to be expected given the criteria for TCP group inclusion. There were no differences in age [amyloid: t_(221)_ = 0.6, *p* = 0.55 and tau: t_(93)_ = 1.0, *p* = 0.32) or sex distribution (amyloid: Fisher's exact *p* = 0.54 and Fisher's exact *p* = 0.48).

**Table 1:** Characteristics of ADNI participants.

**ADNI**	**Amyloid PET sample (*****n*** = **223)**			**Tau PET sample (*****n*** = **95)**		
	**TCP (*****n***=**58)**	**non-TCP (*****n*** = **165)**	***p*** **-value** ^*^	**TCP (*****n*** = **26)**	**non-TCP (*****n*** = **69)**	***p*** **-value** ^*^
Mean age (SD)	72.5 (6.0)	73.0 (6.2)	0.55	70.5 (4.5)	69.4 (5.1)	0.32
Number of females (%)	34 (58.6%)	88 (53.3%)	0.54	18 (69.2%)	41 (59.4%)	0.48
Education: number of years (SD)	17.3 (2.4)	16.3 (2.5)	<0.01^*^	18.0 (1.5)	16.5 (2.3)	<0.005^*^
Mean WMS-R IIA score (SD)	16.6 (1.8)	12.4 (2.6)	<0.0001^*^	17.0 (1.7)	12.4 (3.1)	<0.0001^*^
Mean trails B score (SD)	55.2 (12.2)	87.2 (41.2)	<0.0001^*^	50 (8.5)	78.6 (37.2)	<0.0005^*^
**The 90**+ **study**	**Amyloid PET Sample (*****n*** = **171)**			**Tau PET Sample (n**=**49)**		
	**TCP (*****n*** = **41)**	**non-TCP (*****n*** = **130)**	***p*** **-value** ^*^	**TCP (*****n*** = **13)**	**non-TCP (*****n*** = **36)**	***p*** **-value** ^*^
Mean age (SD)	91.9 (1.5)	92.3 (2.2)	0.20	91.8 (1.4)	91.5 (1.3)	0.53
Number of females (%)	24 (58.5%)	80 (61.5%)	0.85	9 (69.2%)	17 (47.2%)	0.21
Education:			0.90			n/a
High-school graduate or less (%)	6 (14.6%)	21 (16.2%)		4 (30.8%)	6 (16.7%)	
Some college to graduate (%) Some graduate school or higher (%)	18 (43.9%) 17 (41.5%)	58 (44.6%) 51 (39.2%)		4(30.8%) 5 (38.4%)	13 (36.1%) 17 (47.2%)	
Mean CVLT Score (SD)	8.5 (0.5)	6.2 (2.0)	<0.0001^*^	8.5 (0.5)	5.6 (2.0)	<0.0001^*^
Mean Trails B Score (SD)	93.2 (18.4)	156.1 (71.7)	<0.0001^*^	95.9 (16.2)	158.8 (80.4)	0.008

ADNI, Alzheimer's Disease Neuroimaging Initiative; TCP, Top Cognitive Performers; WMS-R, Wechsler Memory Scale-Revised: psychometric; CVLT, California Verbal Learning Test; PET, Positron Emission Tomography. ^*^p-values are for *t*-tests for continuous variables and Fisher's Exact tests for categorical variables.

In *The 90*+ *Study*, there were 171 individuals who fit our inclusion criteria for our amyloid analyses and 49 individuals for tau analyses (Table 1). In contrast to the ADNI cohort, *The 90*+ exhibited no group differences in any demographics (note, the low sample size precluded testing for differences in education for those with tau scans), but did, of course, show the by-definition differences in mean performance in memory [amyloid: t_(169)_ = 7.5, *p* < 0.0001 and tau: t_(47)_ = 5.3, *p* < 0.0001] and executive function [amyloid: t_(169)_ = 5.6, *p* < 0.0001 and tau: t_(47)_ = 2.8, *p* < 0.01].

### Amyloid burden and top cognitive performance across older age groups

**Figure 2:**
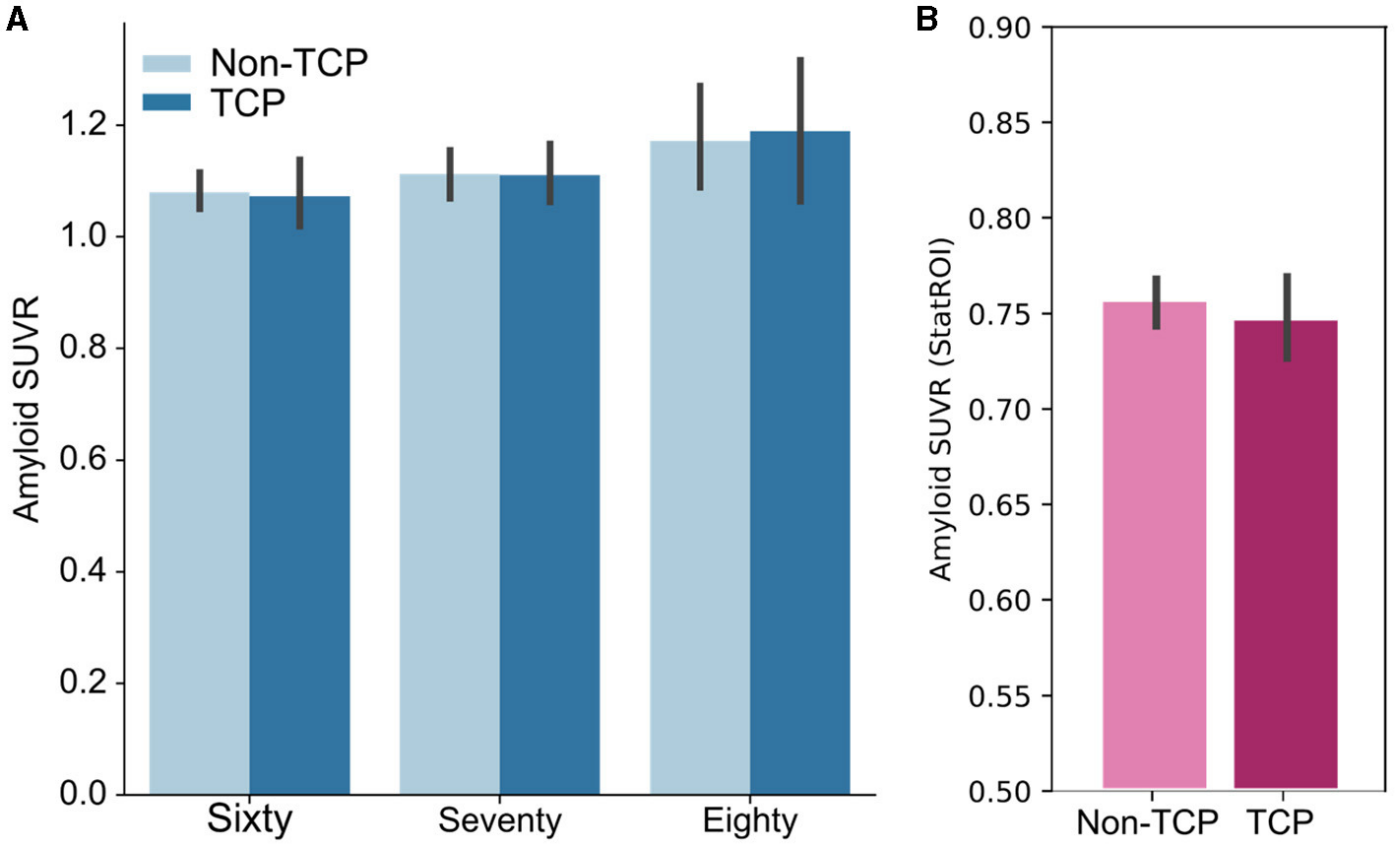
Bar plots show no group differences in amyloid burden across age groups. **(A)** Whole cortex amyloid SUVR measures of TCP vs. non-TCP in sixty, seventy, and eighty year olds from the ADNI dataset. **(B)** StatROI SUVR measures of TCP vs. non-TCP in 90 year old's from The 90+ Study. * Error bars represent 95% confidence intervals.

In the ADNI dataset, 77 of the participants were scored as amyloid-positive (34%). We used an ANCOVA with factors for TCP status, age group (sixty, seventy, eighty), sex, and education to examine amyloid SUVR levels (Figure 2A). After adjusting for age group, sex, and education (Type III sum of squares), the ANCOVA revealed no TCP group differences in amyloid SUVR [F_(1,210)_ = 0.047, *p* = 0.83]. As one might anticipate, after adjusting for the other factors, there was an effect of age group on SUVR [F_(2,210)_ = 5.3, *p* < 0.01] with greater amyloid present in older individuals. However, adjusting for sex and education, results showed no interaction between age group and TCP [F_(2,210)_ = 0.18, *p* = 0.84]. Additionally, we conducted an exploratory analysis to determine if there was any evidence for regional differences in amyloid by age group. For this exploratory analysis, we ran separate ordinary least squares regressions using TCP status, age group, sex, and education for each of the 68 regions, setting an uncorrected alpha threshold of 0.01 to only mildly correct for multiple comparisons in this exploratory analysis. Despite this, none of the regions showed any effect of TCP group status on regional SUVR levels. In The 90+ Study, 73 of the participants were scored as amyloid-positive (43%). As in ADNI, we did not find a difference between TCP and non-TCP in StatROI SUVRs [F_(1,170)_ = 1.04, p=0.43], as shown in Figure 2B.

#### Tau burden and top cognitive performance across older age groups

**Figure 3:**
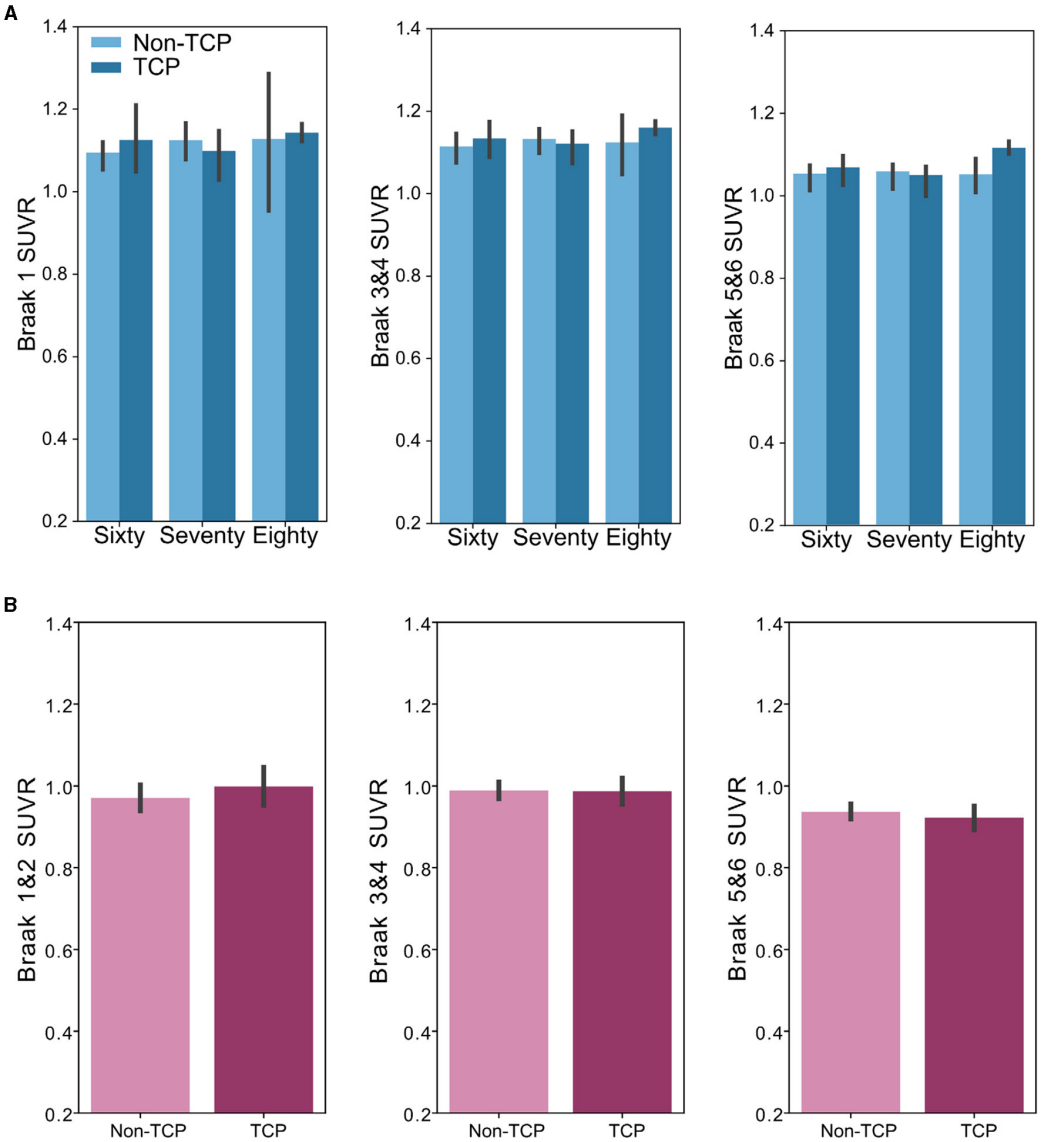
Bar plots show no group differences in Braak region tau burden across age groups. **(A)** Bar plots representing SUVR measures of TCP vs. non-TCP in sixty, seventy, and 80 year olds from the ADNI dataset in the **(left)** Braak 1 ROI, **(middle)** Braak 3 and 4 composite ROI, and **(right)** Braak 5 and 6 composite ROI. **(B):** Bar plots representing SUVR measures of TCP vs. non-TCP in 90 year old's from *The 90*+ *Study* dataset in the **(left)** Braak 1 and 2 composite ROI, **(middle)** Braak 3 and 4 composite ROI, and **(right)** Braak 5 and 6 composite ROI. *Error bars represents Figure 95% confidence intervals.

The same approach was used in the analysis of the tau data, here analyzing the tau load in each Braak region set separately. In the ADNI dataset, ANCOVAs did not reveal group differences in the Braak ROI 1 SUVR [F_(1,94)_ = 0.02, *p* = 0.89], Braak 3&4 composite SUVR [F_(1,94)_ = 0.43, *p* = 0.51], or Braak 5&6 composite SUVR [F_(1,94)_ = 0.84, *p* = 0.36] after accounting for age, sex, and education, as shown in Figure 3. Unlike in measures of amyloid, there was no effect of age group on any of the Braak ROI SUVRs (all p's>0.1). Additionally, there were no interactions of age group by TCP in each SUVR (all p's>0.2). Similarly, data from *The 90*+ *Study* showed no differences in Braak ROI 1&2 composite SUVR [F_(1,47)_ = 0.02, *p* = 0.90], Braak 3&4 composite SUVR [F_(1,47)_ = 0.07, *p* = 0.78], or Braak 3&4 composite SUVR [F_(1,47)_ = 0.47, *p* = 0.50].

## Discussion

Our goal was to assess whether TCPs, a cohort of successful aging individuals, exhibited group differences in Alzheimer's disease-related pathology when compared to their peers to determine whether their high level of cognitive performance might be attributed to a lack of pathology (resistance) or maintained cognition despite pathology (resilience). In line with the majority of the current literature, our results support the hypothesis that TCP individuals do not exhibit reduced amyloid or tau relative to their peers, a relationship that persist in the oldest-old. These data suggest that intact cognitive performance does not reflect resistance to AD-related pathology, but is more consistent with resilience to the pathology.

Here, we tried to broaden the scope to both larger populations and to wider age ranges to help address the small sample sizes in these reports. In our primary analysis, we sought to use the standardized *a priori* composite SUVR measures rather than voxelwise analyses of specific meta ROIs (inferior temporal, precuneus, entorhinal cortex, middle occipital, and orbitofrontal) used in the only other tau study (Hoenig et al., 2020) or specific ROIs derived from the analyses. As our goal was to determine whether TCP was a result of resilience to AD-related pathology, using these *a priori* regions that are used in AD diagnosis was the clearer analysis path, but does leave open the possibility of more regionally-specific amyloid or tau differences being related to TCP.

That said, we did perform an exploratory analysis to address this, at least in the case of amyloid, where regional data were available. Baran and colleagues (Baran et al., 2018) were able to show that though their Supernormals did not exhibit any differences in whole cortical amyloid, there was a difference in the isthmus cingulate following a 68 ROI analysis across the cortex. They noted, though, that since the isthmus cingulate is a small ROI and their FreeSurfer segmentations were done manually, their analysis was ‘vulnerable to subjectivity in the correction of topological defects' (Baran et al., 2018). Our analysis revealed no differences in any of the age groups in amyloid burden, suggesting that even regionally, amyloid is not associated with TCP.

### Limitations

We acknowledge several limitations on the generalizability of the results presented here. It is important to note the role of volunteer and selection biases in these analyses. People who are able and willing to participate in imaging tend to be healthier and meet *a priori* selection criteria. One large study examining the nature of volunteer and selection biases found that those who were more likely to participate in studies were also more likely to be cognitively healthy, well-educated, and male compared to their counterparts who were not interested in participating (Ganguli et al., 2015). It is possible that having such biases might mask the true extent of differences in AD pathology between TCP and non-TCP due to the potential of having overall healthier individuals in both groups. In addition to such typical biases, given that participants from *The 90*+ *Study* are largely survivors of Leisure World Cohort Study and recruited from a retirement community in Laguna Woods, California, it is certainly possible that participants are not fully representative of the population. *The 90*+ *Study* participants tend to be mostly Caucasian and of both high socioeconomic and education status. We also acknowledge that the lack of a standard successful aging definition makes comparisons between studies difficult. Given the nature of data availability in ADNI and *The 90*+ *Study*, even we had to use different measures of delayed memory. As previously mentioned, the WMS-R IIA and CVLT were chosen as tests of delayed recall to mirror SuperAging standard as closely as possible, limited by what data is available in each dataset.

Our results are also cross-sectional and only represent a snapshot of an individual's cognition and pathological profile. One successful aging study that did include longitudinal data found that while amyloid burden did not differ between Harvard Aging Brain Study's optimal and typical performers, those who maintained their cognition over 3 years (e.g., maintainers) displayed lower amyloid burden at baseline when compared to those that did not. Dekhtyar and colleagues suggested that individuals who did not maintain their optimal performance may be representative of a preclinical trajectory (Dekhtyar et al., 2017). Preclinical Alzheimer's disease, first described as cognitively normal individuals that exhibit pathology at autopsy, is the stage before diagnosis and noticeable symptoms appear (Hubbard et al., 1990; Dubois et al., 2016). Longitudinal studies show amyloid deposition measured by PET 15 years before symptom onset (Bateman et al., 2012). Thus, it is possible that since we are studying baseline visits, we are capturing individuals on the preclinical trajectory to Alzheimer's disease or that the rate of accumulation may be able to resolve an effect.

Much like the aging population, The 90+ Study has revealed that participants can experience varying conditions in addition to Alzheimer's disease, reflecting the intricate nature of health in the oldest-old. For example, a post-mortem study examining 90+ year old Superior global cognitive performers (SGCP) included individuals with histologically confirmed atherosclerosis, Lewy Body Disease (LBD), Hippocampal sclerosis (HS), and Limbic-predominant age-related TDP-43 encephalopathy (LATE) (Biswas et al., 2023). The 90+ Study has also shown that higher baseline white matter hyperintensity volumes were associated with worse scores in global cognition, episodic memory, and executive functioning tasks (Legdeur et al., 2019). White matter hyperintensities are thought to be the result of cerebral small vessel disease and may account for increased blood brain barrier permeability and demyelination seen in the aging brain (Haller et al., 2013). Other possible co-morbidities can include cardiovascular diseases, hypertension, and diabetes. Additionally, these individuals commonly face challenges related to frailty. For example, frailty was observed in 24% of individuals aged 90 to 94, while the prevalence increased to 39.5% among those aged 95 and above (Lee et al., 2016). Therefore, despite their normal cognition, participants in this study can exhibit a range of other health conditions, demonstrating the complex nature of aging and the multifaceted health challenges faced by the oldest-old population.

Finally, we should note that our results are centered on a lack of a difference between these two groups. Almost without question, there are other, non-AD-specific pathologies and there are uncontrolled for compensatory mechanisms would induce variance that would weaken any potential relationship between TCP and AD pathology. We cannot speak to how aspects of “brain reserve” or “brain maintenance” (Stern et al., 2023) outside of amyloid and tau might impact this. For example, a previous post-mortem study (*n* = 5) showed that though there were no observed group differences in total neuronal size or count, 80+ year old SuperAgers displayed a higher density of von Economo neurons (VENs) when compared to elderly controls and those with MCI (Gefen et al., 2015). Similarly Harrison et al. (2018) found that successful agers displayed lower white matter hypointensity volumes in comparison to other typical older adults. These are associated with aging (Wei et al., 2019) and their lower presence could suggest better resistance in older adults. Thus, while there may be other markers of reduced pathology or even enhancements, with respect to amyloid and tau, the clear pathologies associated with Alzheimer's, their presence at typical rates in TCP, supports the parsimonious explanation being one of resilience to pathology rather than resistance to its development.

### Summary

In this study, we sought to determine whether older adults who exhibit exceptionally high levels of cognitive performance despite their advancing age might be maintain this via resistance to the accumulation of amyloid and tau. Data from both ADNI and The 90+ Study were remarkably consistent in showing no evidence whatsoever in support of this hypothesis. Neither amyloid nor tau levels, be it in traditional AD-related regions or in whole-brain analyses showed any ability to discriminate TCP performance. This suggests that older adults who maintain such high levels of cognitive performance do so despite entirely typical development of amyloid and tau, not by avoiding their buildup.

## Data availability statement

The original contributions presented in the study are included in the article/Supplementary material, further inquiries can be directed to the corresponding author.

## Ethics statement

The studies involving humans were approved by UC Irvine IRB & ADNI joint IRB. The studies were conducted in accordance with the local legislation and institutional requirements. The participants provided their written informed consent to participate in this study.

## Author contributions

ED: Conceptualization, Formal analysis, Investigation, Methodology, Visualization, Writing – original draft, Writing – review & editing. MC: Funding acquisition, Methodology, Project administration, Resources, Supervision, Writing – original draft, Writing – review & editing. CK: Funding acquisition, Methodology, Project administration, Resources, Supervision, Writing – original draft, Writing – review & editing. CS: Conceptualization, Data curation, Formal analysis, Funding acquisition, Investigation, Methodology, Project administration, Resources, Supervision, Validation, Visualization, Writing – original draft, Writing – review & editing.
