# Supplementary material for: Resilience to AD pathology in Top Cognitive Performers
Source: Front Aging Neurosci. 2024 Jul 11;16:1428695. doi: 10.3389/fnagi.2024.1428695 (PMC11270559; doi:10.3389/fnagi.2024.1428695)
Supplement: Supplementary file 3 [file Data_Sheet_3.DOCX]

# ADNI.csv

This file contains the subject IDs used and the TCP status for the ADNI participants used in this study. Specific data must be downloaded from ADNI directly and cannot be shared here.

- PTID_amyloid: ADNI IDs of those used in the amyloid PET analyses

- TCP_amyloid: Conjunction of WMS and Trails-B top 50% used to define Top Cognitive Performer (TCP) status. 1=TCP, 0=Non-TCP

- AgeGroup_amyloid: Age decade in the amyloid analysis

- PTID_tau: ADNI IDs of those used in the tau PET analyses

- TCP_tau: Conjunction of WMS and Trails-B top 50% used to define Top Cognitive Performer (TCP) status. 1=TCP, 0=Non-TCP

- AgeGroup_tau: Age decade in the tau analysis

# 90Plus.csv

This file contains the data from the amyloid and tau PET scans used to make Figures 2B and 3B

- ID: ID code - simple 1-N

- Gender: 0=Male, 1=Female

- Education: 1=Did not complete 8th grade, 2=Some high school, 3=High school graduate, 4=Vocational school, 6=Some college, 7=College graduate, 8=Some graduate school, 9=Advanced degree

- CVLD_LDelay: California Verbal Learning Test Long Delay score

- DigitsBackwards: Digit-span (reversed) score

- BNTc: Boston Naming Task score

- TRLBtime: Time to complete the Trails-B task

- TCP: Conjunction of CVLT and Trails-B top 50% used to define Top Cognitive Performer (TCP) status. 1=TCP, 0=Non-TCP

- StatROI: PET amyloid (AV45) summary SUVR (standardized uptake value ratio): This is quantified slightly differently in 90+ than in ADNI as the SUVR of posterior cingulate and precuneus regions relative to an eroded cerebral white matter mask as a reference region.

- AV1451_Braak1-2_WM AV1451 mean SUVR (based on white matter ref) over Braak regions 1-2

- AV1451_Braak3-4_WM AV1451 mean SUVR (based on white matter ref) over Braak regions 3-4

- AV1451_Braak5-6_WM AV1451 mean SUVR (based on white matter ref) over Braak regions 5-6
